# Diagnostic Challenges in Seronegative Celiac Disease: A Case of Massive Splenomegaly and Persistent Metabolic Imbalance

**DOI:** 10.7759/cureus.100816

**Published:** 2026-01-05

**Authors:** Mathab Adam, Ahmed H Ahmed, Aia A Ibrahim, Amira Nabil Mohammed Mansour, Elhassan Abdullah, Muhammad Zahid, Khalid Azhar

**Affiliations:** 1 Internal Medicine, Hamad Medical Corporation, Doha, QAT; 2 Internal Medicine , Hamad Medical Corporation, Doha, QAT; 3 Radiology, Hamad Medical Corporation, Doha, QAT; 4 Medicine, Hamad Medical Corporation, Doha, QAT

**Keywords:** case report, celiac disease, electrolyte disturbances, malabsorption, seronegative celiac disease, splenomegaly

## Abstract

We report the case of a young female with recurrent and severe electrolyte disturbances, including persistent hypokalemia, hypocalcemia, hypomagnesemia, and metabolic acidosis, in association with splenomegaly and chronic abdominal complaints. Despite extensive investigations, including repeated biochemical testing, coagulation studies, comprehensive imaging, and a broad infectious and autoimmune work-up, no clear etiology was initially identified. Notably, celiac serology was negative, while stool studies, hepatitis and HIV serology, tuberculosis screening, and tumor markers were unremarkable. Her medical, surgical, and family histories were unremarkable, with no evidence of chronic illness or malignancy. Given the constellation of clinical, laboratory, and imaging findings, seronegative celiac disease (CD) was considered the most likely diagnosis, although not definitively proven. initiation of a strict gluten-free diet and supportive management, with progressive normalization of electrolyte disturbances, significant symptomatic relief, underscoring the importance of early recognition and appropriate treatment of seronegative CD. This case highlights the diagnostic challenges of suspected seronegative CD; the importance of correlating clinical, laboratory, and imaging features; and the need to maintain a high index of suspicion in atypical presentations.

## Introduction

Celiac disease (CD) is an immune-mediated enteropathy triggered by the consumption of dietary gluten in genetically susceptible individuals. The global prevalence is estimated at 1-2% [1,2]. However, due to the diversity of clinical manifestations, which include atypical and extraintestinal symptoms, many cases remain undiagnosed. The mainstay of diagnosis is serologic testing paired with duodenal biopsy. Serologic assays (such as anti-tissue transglutaminase (tTG) and anti-endomysial antibodies (EMA)), although considered highly sensitive in overt cases with total villous atrophy on biopsy (>90%), have significantly reduced sensitivity in partial or early mucosal disease, leading to false-negative results [1,2].

This diagnostic limitation explains the phenomenon of seronegative celiac disease (SNCD), in which patients lack serologic markers despite the presence of histologic and genetic evidence of CD [3]. Its recognition remains a clinical challenge, with guidelines emphasizing concordance of duodenal biopsy findings, HLA genotyping (HLA-DQ2 or DQ8 positivity), and clinical response to a gluten-free diet (GFD) in such cases [4]. HLA genotyping was not performed, as the test requires costly external outsourcing, which was not feasible for the patient, who subsequently declined further evaluation.

CD manifests across a wide clinical spectrum. The classic presentation includes gastrointestinal symptoms such as chronic diarrhea, abdominal pain, bloating, weight loss, and steatorrhea, while others may exhibit extraintestinal features including iron-deficiency anemia, osteoporosis, dermatitis herpetiformis, neurological symptoms, and menstrual disturbances [5]. Splenomegaly is an unusual presentation and may mimic hematologic malignancies or portal hypertension on imaging. One unique report describes the incidental detection of CD on FDG-PET/CT, characterized by focal duodenal uptake and splenomegaly, both of which resolved after initiating a GFD, highlighting the potential for atypical imaging findings [6]. Reports of SNCD with massive splenomegaly and profound electrolyte disturbances remain exceedingly scarce in the literature, making this case a noteworthy diagnostic dilemma. Accordingly, this case is presented to underscore the diagnostic complexity of SNCD, particularly when it presents with uncommon features such as massive splenomegaly and severe metabolic derangements, including persistent hypokalemia and hypocalcemia. It further highlights the importance of maintaining a high index of suspicion in atypical cases, where timely recognition and dietary intervention can prevent complications, correct nutritional deficiencies, and significantly improve quality of life.

## Case presentation

A 41-year-old Egyptian woman from Al Fayoum, with a seven-year history of intermittent watery diarrhea and recurrent hypocalcemia, hypomagnesemia, hypokalemia, and hypoalbuminemia, presented with abdominal pain, vomiting, diarrhea, and profound weakness. On admission, her blood pressure was 120/70 mmHg, heart rate was 80 beats per minute, respiratory rate was 16 per minute, and oxygen saturation was 99% on room air.

She appeared cachectic and pale, but was afebrile and hemodynamically stable. Abdominal examination revealed a distended, soft abdomen with a markedly enlarged spleen palpable well below the costal margin, consistent with massive splenomegaly. There was no clinically appreciable hepatomegaly, lymphadenopathy, or stigmata of chronic liver disease. Neurological examination was intact apart from carpopedal spasms, consistent with hypocalcemia.

Laboratory evaluation showed microcytic anemia with low serum iron, reduced transferrin saturation, and high-normal TIBC, consistent with combined iron deficiency and anemia of inflammation (Table 1).

**Table 1:** Hematology

Parameter	10/07/25	1/08/25	17/08/25	Reference range
Hemoglobin (g/dL)	10.6 (L)	10.6 (L)	13.8	12.0–15.0
RBC (x10^6^/uL)	4.0	4.0	5.3 (H)	3.8–4.8
Platelets (x10^3^/uL)	132 (L)	116 (L)	111 (L)	150–410
WBC (x10^3^/uL)	4.5	5.0	12.5 (H)	4.0–10.0

Despite marked hypoalbuminemia, liver enzymes and other hepatic synthetic markers remained within normal limits, supporting a malabsorptive etiology rather than intrinsic hepatic dysfunction. Persistent electrolyte derangements included low corrected calcium, magnesium, potassium, phosphorus, and low vitamin D (Table 2).

**Table 2:** Blood chemistry

Parameter	07/10/25	11/07/25	12/07/25	11/08/25	15/08/25	18/08/25	19/08/25	Reference range
Sodium (mmol/L)	137	-	135	135	134	132	133	133–146
Potassium (mmol/L)	2.9 (L)	4.1	3.9	2.1 (L)	2.9 (L)	3.2 (L)	NA/hemolyzed	3.5–5.3
Chloride (mmol/L)	108	-	109 (H)	108	113 (H)	105	103	95–108
Bicarbonate (mmol/L)	18 (L)	-	16 (L)	16 (L)	9 (L)	16 (L)	19 (L)	22–29
Calcium adjusted (mmol/L)	1.95 (L)	2.17 (L)	2.16 (L)	2.14 (L)	2.22	—	—	2.20–2.60
Phosphorus (mmol/L)	-	-	0.64 (L)	0.54 (L)	-	-	-	0.80–1.50
Magnesium (mmol/L)	0.69 (L)	0.68 (L)	0.84	1.01 (H)	0.73	0.78	-	0.70–1.00
Creatinine (µmol/L)	54	-	52	78	78	59	56	44–80
Urea (mmol/L)	3.0	-	4.3	2.9	1.8 (L)	1.5 (L)	1.1 (L)	2.5–7.8
Albumin (g/L)	22 (L)							35-50

Venous blood gas demonstrated a non-anion gap metabolic acidosis with low bicarbonate and a marked base deficit (Table 3).

**Table 3:** Blood gases

Parameter	10/07/25	11/08/25	13/08/25	15/08/25	19/08/25	Reference range
pH	7.38	7.32	7.19	7.19	7.43	7.32–7.42
pCO2 (mmHg)	33 (L)	31 (L)	25 (L)	26 (L)	35 (L)	41–51
pO2 (mmHg)	76 (H)	62 (H)	83 (H)	99 (H)	70 (H)	25-40
HCO3 (mmol/L)	19.5 (L)	16.0 (L)	9.5 (L)	9.9 (L)	23.2	23–29
Lactate (mmol/L)	0.70	0.90	0.50	0.30 (L)	0.4 (L)	0.5–2.2

Her coagulation profile demonstrated intermittent derangements, with prolonged PT/INR and elevated aPTT during admission, though fibrinogen and D-dimer remained within normal limits (Table 4).

**Table 4:** Coagulation profile

Parameter	23/07/25	15/08/25	17/08/25	18/08/25	Reference Range
PT (seconds)	16.2 (H)	37.2 (H)	39.1 (H)	12.8 (H)	9.4–12.5
INR	1.5	3.4	3.5	1.2	0.8–1.2
APTT (sec)	31.1	40.5 (H)	54.4 (H)	32.9	25.1–36.5
Fibrinogen (g/L)	-	-	3.70	-	2.00–4.10
D-dimer (mg/L FEU)	-	-	0.46	-	0.00–0.49

Renal function remained preserved. The nephrology team was consulted; urine electrolytes were unremarkable, and the acidosis was attributed to diarrheal losses (Table 5).

**Table 5:** Urine

Parameter	11/07/25	2025-07-12	Reference range
24-h calcium (mmol/24h)	-	3.5	2.5–7.5
24-h sodium (mmol/24h)	-	149	40–220
Urine protein/Cr ratio (mg/mmol)	134.26 (H)	-	<20
Urine albumin ratio (mg/mmol)	5.4 (H)	-	0.0–3.5

Infectious screening, including hepatitis B and C, HIV, EBV, CMV, *Brucella*, and *Leishmania* serologies, was negative (Table 6).

**Table 6:** Infectious workup

Lab test	Findings
*Brucella* Ab IgG	Nonreactive
*Brucella* Ab IgM	Nonreactive
*Leishmania* Ab	Negative
*Schistosoma* Ab	Negative
CMV Ab IgM	Nonreactive
CMV PCR	Not detect
Hepatitis B core Ab	Nonreactive
Hepatitis B surface Ab	Nonreactive
Hep Bs Ab Num	<2.00
Hepatitis B surface antigen	Nonreactive
Hepatitis C Ab	Nonreactive
HIV Ag/Ab Combo	Nonreactive
Stool culture	No *Salmonella*, No *Shigella*
Blood culture aerobic	No growth
Blood culture anaerobic	No growth
Ova + parasites stool	No ova or parasites seen. *Giardia/Cryptosporidium* antigen not detected

Autoimmune serologies, including ANA, dsDNA, ANCA, anti-thyroid antibodies, and autoimmune liver markers (AMA, ASMA), were unremarkable. Serum immunoglobulins and total IgA were within normal limits. IgG anti-tTG antibodies were negative. Importantly, the patient was not on a gluten-free diet at the time of serologic testing, excluding dietary gluten restriction as a cause of negative celiac serology.

Abdominal tuberculosis was specifically considered given regional epidemiology; however, TB screening, including Quantiferon-TB Gold, stool AFB, chest X-ray, CT abdomen, stool studies, and duodenal biopsies, showed no evidence of granulomas, acid-fast bacilli, or radiologic features of peritoneal TB.

**Figure 1:**
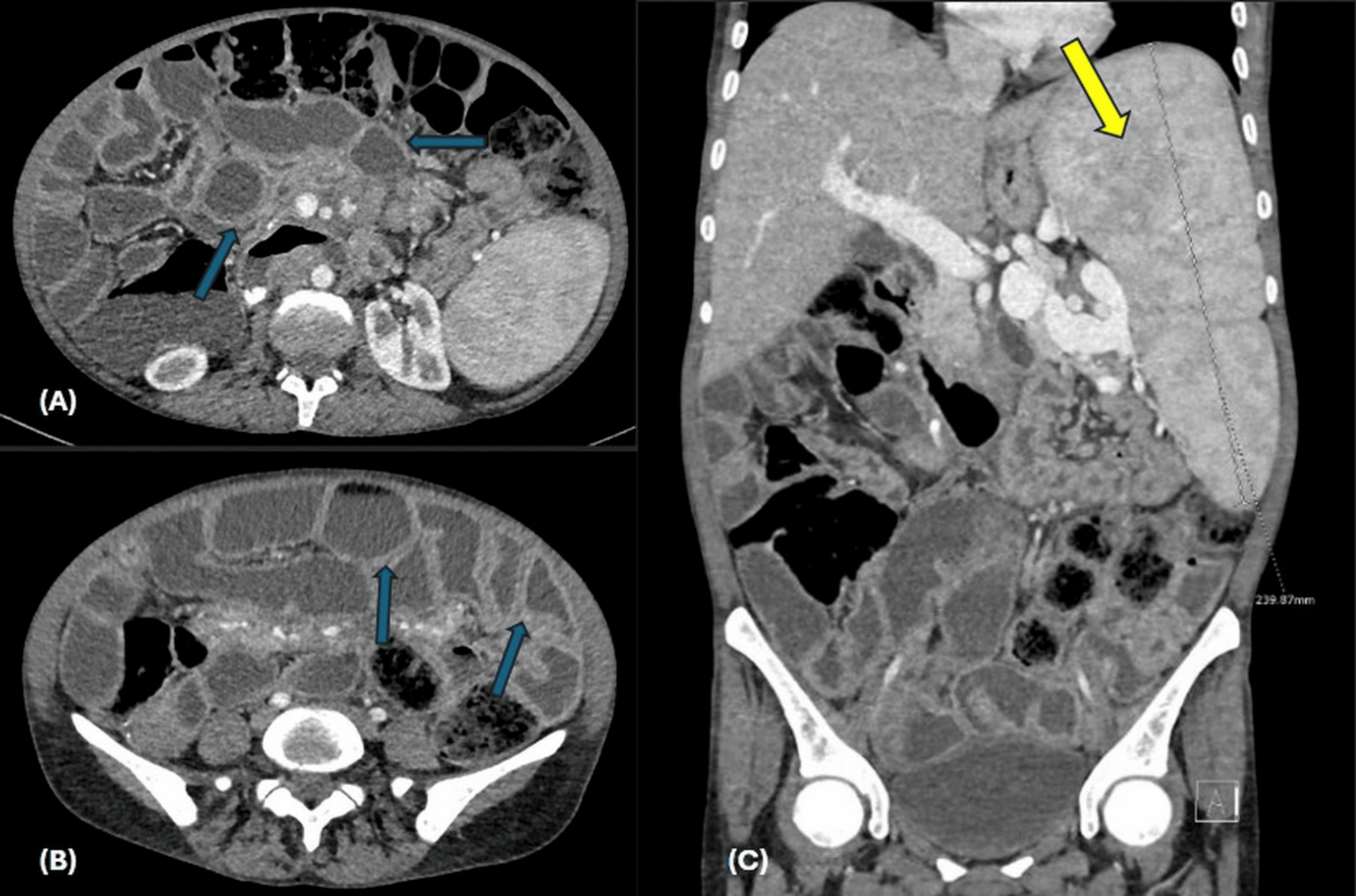
Contrast-enhanced CT enterography Axial images (A) and (B) and a coronal image showing diffuse circumferential mural small bowl wall thickening (blue arrows), as well as an enlarged spleen measuring 24 cm (yellow arrow).

**Figure 2:**
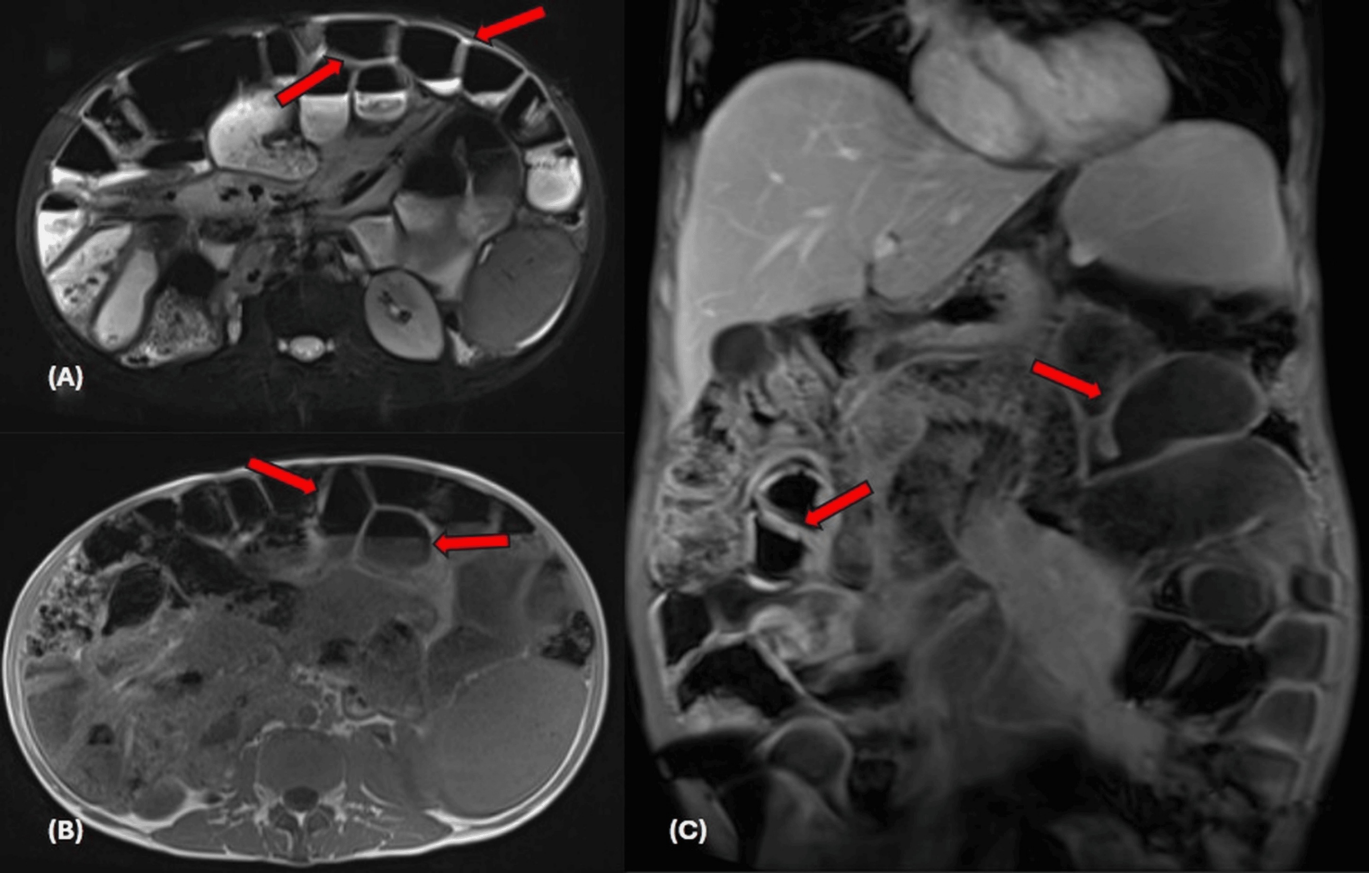
MRI abdomen with contrast Axial T2 image (A), axial post-contrast T1, and coronal T1 post-contrast, showing diffuse circumferential small bowel thickening.

Cross-sectional imaging, including CT enterography and MRI abdomen (Figures 1-2), confirmed massive splenomegaly up to 25 cm, with mild hepatomegaly, diffuse small-bowel thickening, mesenteric lymphadenopathy encasing mesenteric vessels, and small ascites.

**Figure 3:**
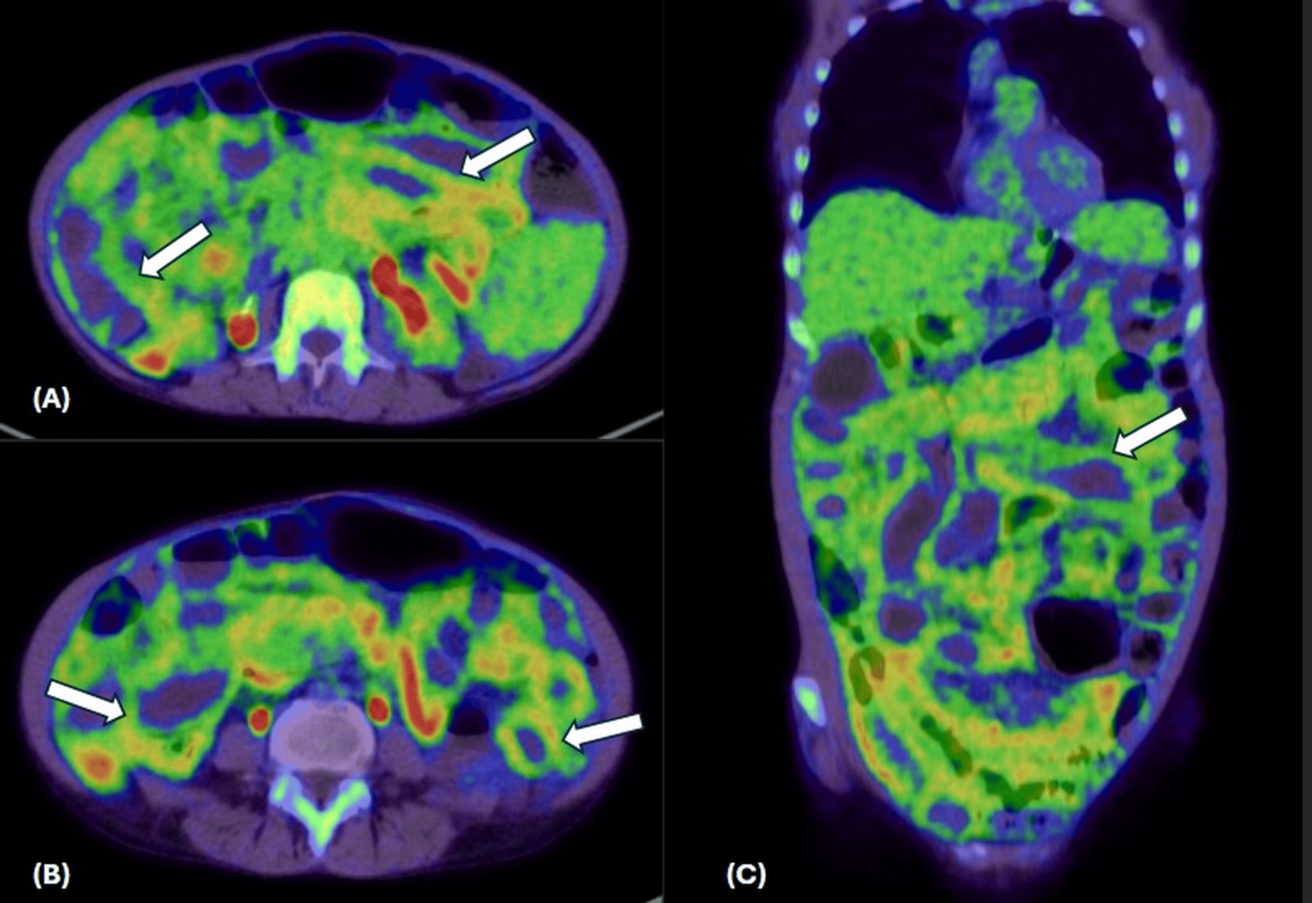
FDG PET/CT scan Axial cuts (A), (B), and coronal cuts of fused PET/CT images showing thickened small bowel with avid FDG uptake (white arrows).

PET/CT demonstrated diffuse bone marrow hypermetabolism with heterogeneous splenic uptake and mild mesenteric activity, without focal FDG-avid lesions (Figure 3).

Endoscopic evaluation using esophagogastroduodenoscopy (OGD) revealed normal esophageal mucosa and a normal-appearing gastric fundus, body, and antrum. The duodenal mucosa showed mucosal nodularity and erythema with flattening of the duodenal folds in the first and second parts of the duodenum. During push enteroscopy, the scope was advanced to the proximal jejunum. The gastric mucosa appeared slightly atrophic, while the small bowel mucosa, extending from the duodenum to the proximal jejunum, was diffusely edematous with continuous loss of villous pattern, scalloping, and cobblestoning. Colonoscopy was unremarkable. Histological examination of duodenal and jejunal biopsies showed preserved villous architecture with a mild increase in intraepithelial lymphocytes (50-60 per 100 enterocytes), consistent with Marsh I-II changes. No parasites, granulomas, dysplasia, or malignant features were observed. Flow cytometry performed on both endoscopic biopsy specimens and peripheral blood revealed no evidence of clonal lymphoid populations.

A wide differential diagnosis was considered, given the severity and chronicity of the patient’s symptoms. Colonoscopy with biopsies and stool calprotectin were performed to evaluate for inflammatory bowel disease, all of which were normal. An autoimmune panel comprising ANA, dsDNA, ANCA, anti-thyroid antibodies, total IgA, and autoimmune liver antibodies was obtained to assess for immune-mediated enteropathies; all results were negative. Whipple’s disease was excluded through duodenal biopsies that demonstrated no PAS-positive macrophages. Lymphoproliferative and infiltrative disorders, including enteropathy-associated T-cell lymphoma, were evaluated using CT imaging, peripheral smear, LDH, and histologic examination of duodenal biopsies, none of which showed malignancy or atypical lymphoid populations. Portal-hypertension-related hypersplenism was ruled out through abdominal ultrasound and CT, which revealed normal liver morphology, normal portal vein diameter, and absence of ascites. Pancreatic exocrine insufficiency was assessed through serum lipase and amylase levels, along with abdominal imaging, all of which were unremarkable.

During admission, electrolyte disturbances were managed with parenteral replacement before transitioning to high-dose oral therapy. She also received calcitriol, sodium bicarbonate, thiamine, and multivitamin supplementation. A gluten-free diet was initiated during hospitalization, with dietitian referral for counseling. While she continued to experience recurrent hypokalemia and metabolic acidosis, her diarrhea improved substantially with dietary modification.

At discharge, the patient showed clinical improvement, though she remained cachectic, with stabilized electrolyte levels maintained through oral supplementation. She was discharged on potassium, calcium, magnesium, bicarbonate, and loperamide, along with strict gluten-free dietary instructions. A subsequent bone marrow biopsy revealed normocellular marrow with no evidence of malignancy or clonal lymphoid populations. Following adherence to a gluten-free diet, the patient experienced significant symptomatic improvement. During a telephonic follow-up conducted several weeks post-discharge, she reported near-complete resolution of diarrhea and continued compliance with dietary recommendations.

In summary, this patient presented with severe malabsorption, refractory electrolyte abnormalities, and massive splenomegaly. The combination of chronic diarrhea, Marsh I-II histological changes, negative celiac serology, and clinical improvement on a gluten-free diet is highly suggestive of SNCD. The persistent marrow hypermetabolism and splenic uptake raised concern for an indolent lymphoproliferative disorder; however, repeated mucosal biopsies with flow cytometry, peripheral blood analysis, and bone marrow biopsy have thus far excluded overt lymphoma.

## Discussion

SNCD is a diagnostically challenging variant of CD, where serologic markers can be negative in up to 15% of biopsy-proven cases [7,8]. Among patients with seronegative enteropathy, approximately one-third to one-half are ultimately diagnosed with SNCD [9]. Our patient presented with severe malabsorptive symptoms, persistent metabolic disturbances, and splenomegaly, an unusual constellation that prompted extensive investigation. Despite classical histological findings of villous atrophy, the absence of serologic markers and the unavailability of HLA genotyping initially complicated the diagnostic process.

Compared with seropositive CD, SNCD tends to present later in life and with more severe malabsorptive features, including diarrhea, weight loss, and micronutrient deficiencies [10]. While splenic hypofunction is a well-established extraintestinal manifestation of CD [11], frank splenomegaly is exceedingly rare. There is precedent for such atypical presentations in the literature. Nevertheless, splenomegaly can occur in association with CD, most often in the setting of longstanding disease, portal hypertension, or immune dysregulation. For example, Panda et al. [6] reported a patient with biopsy-confirmed CD and PET/CT-detected splenomegaly initially mimicking lymphoma, which improved following a gluten-free diet. Likewise, Zamani et al. [12] described a 54-year-old man with CD who developed idiopathic (non-cirrhotic) portal hypertension, manifesting as massive splenomegaly, large-volume ascites, and hypersplenism. Notably, the portal hypertension and splenomegaly in that patient improved after the introduction of a gluten-free diet, implicating active CD as a trigger for these complications. This parallel experience reinforces our own findings and suggests that the intense immune activity associated with CD can underlie splenic enlargement in susceptible individuals. We postulate an immune-mediated mechanism for the splenic enlargement, given CD's profound immunologic activation [13]. Although rare, these cases confirm that splenomegaly falls within the broader clinical spectrum of celiac-associated complications. The absence of HLA testing in this patient represents a limitation, as the presence of DQ2 or DQ8 haplotypes would have further supported the diagnosis. However, current clinical guidance permits a presumptive diagnosis of SNCD when serology is negative, histology is consistent, other causes are excluded, and a clinical response to a gluten-free diet is observed. This case fulfills these criteria and reinforces the value of therapeutic gluten withdrawal as both a diagnostic and treatment tool in suspected SNCD [10].

The differential diagnosis of seronegative enteropathy includes autoimmune enteropathy, infectious etiologies such as giardiasis or Whipple’s disease, inflammatory bowel disease, drug-induced enteropathy, and small-bowel lymphoma [4]. A broad workup was done for our patient to exclude these differentials. Stool examinations and cultures revealed no pathogens. Serologies for HIV, tropical infections, and abdominal tuberculosis were negative. Imaging showed no evidence of Crohn’s disease, crypt apoptosis, or occult malignancy. Immunoglobulin levels were normal (arguing against significant antibody deficiency or common variable immunodeficiency), and there was no history of medications known to cause enteropathy. Autoimmune enteropathy, typically accompanied by other autoimmune features and anti-enterocyte antibodies, was considered unlikely given the major remission on GFD. This distinction is supported by Akram et al. [14], emphasizing that autoimmune enteropathy is largely unresponsive to dietary therapy and often requires immunosuppressive treatment. The presence of massive splenomegaly and diffuse bone marrow uptake initially raised concern for an indolent lymphoproliferative disorder; however, repeated flow cytometry and bone marrow examination revealed no evidence of hematologic malignancy. Given the well-documented association of CD with hematologic abnormalities, including IgA deficiency and an increased risk of lymphoma [15], a thorough evaluation was necessary. The absence of any alternative explanation, combined with the biopsy findings and clinical context, left gluten-driven enteropathy as the most plausible diagnosis. Strict dietary gluten exclusion led to substantial clinical recovery, a response that competing diagnoses would not be expected to show, thereby solidifying the diagnosis of SNCD.

From a prognostic standpoint, SNCD has been associated with slower mucosal recovery and an increased risk of complications, including refractory CD and enteropathy-associated T-cell lymphoma [16,17]. These observations emphasize the need for strict dietary adherence and long-term surveillance.

This case is notable for the coexistence of seronegative enteropathy and massive splenomegaly, a rare but documented association in the literature. Although the underlying mechanism remains uncertain, potential contributors include chronic antigenic stimulation, immune dysregulation within the reticuloendothelial system, or altered portal hemodynamics. Recognizing this relationship is clinically relevant; the presence of splenomegaly should not deter clinicians from considering SNCD when histologic and clinical findings support the diagnosis despite negative serology.

## Conclusions

This case highlights the diagnostic complexity of SNCD, particularly when atypical features such as persistent metabolic derangements and massive splenomegaly are present. While splenomegaly is not commonly described in the conventional literature on CD, isolated reports, such as Panda et al. and Zamani et al., have documented this rare association mediated by secondary mechanisms. In our case, a thorough diagnostic work-up, including negative autoimmune serologies, normal imaging, unremarkable colonoscopy, exclusion of infectious and infiltrative diseases, and a negative bone marrow biopsy, helped rule out alternative etiologies. Due to limited access and cost limitations, HLA genotyping was not carried out; nonetheless, the diagnosis was strongly supported by the distinctive histologic findings and substantial clinical improvement on a gluten-free diet. This case reinforces the importance of maintaining diagnostic vigilance in atypical seronegative presentations, where timely recognition and dietary intervention can help prevent progression to refractory disease or lymphoproliferative complications.
